# Application of a high resolution T1 mapping with MOLLI (hrMOLLI) in patients in clinical setting: a reproducibility study

**DOI:** 10.1186/1532-429X-14-S1-O82

**Published:** 2012-02-01

**Authors:** Ana Pastor, Zhong Chen, Tobias Voigt, Tobias Schaeffter, Reza Razavi, Eike Nagel, Valentina O Puntmann

**Affiliations:** 1 Cardiovascular Imaging, King's College London, London, UK; 2 Philips Healthcare, London, UK

## Summary

Several methodologies were proposed to evaluate myocardial T1 values by cardiovascular magnetic resonance based on Gadolinium contrast enhancement. Whereas potentially valuable for evaluation of myocardial fibrosis, none of these methodologies have been sufficiently robust and consistently translated into clinical routine. We propose that imaging with a high-resolution modified Look-Locker inversion recovery sequence (hrMOLLI) of native and post-contrast myocardium can provide a robust tool in the clinical setting.

## Background

Myocardial fibrosis is associated with myocardial dysfunction and remodelling leading to adverse outcomes. We propose that imaging with a high-resolution modified Look-Locker inversion recovery sequence (hrMOLLI) of native and post-contrast myocardium can provide a robust tool in the clinical setting.

## Methods

**Figure 1:**
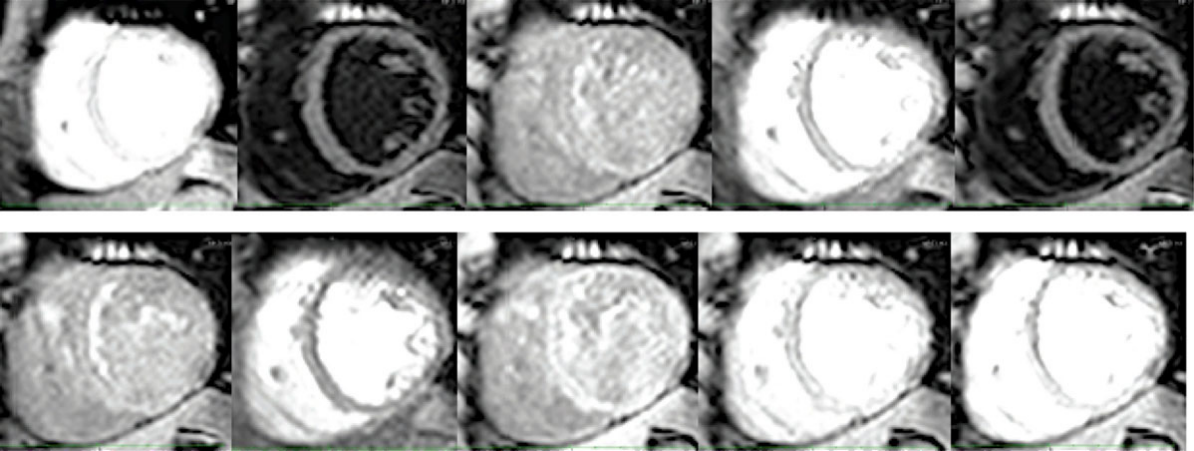
Representative images of post-contrast MOLLI sequence (11th phase omitted).

**Figure 2:**
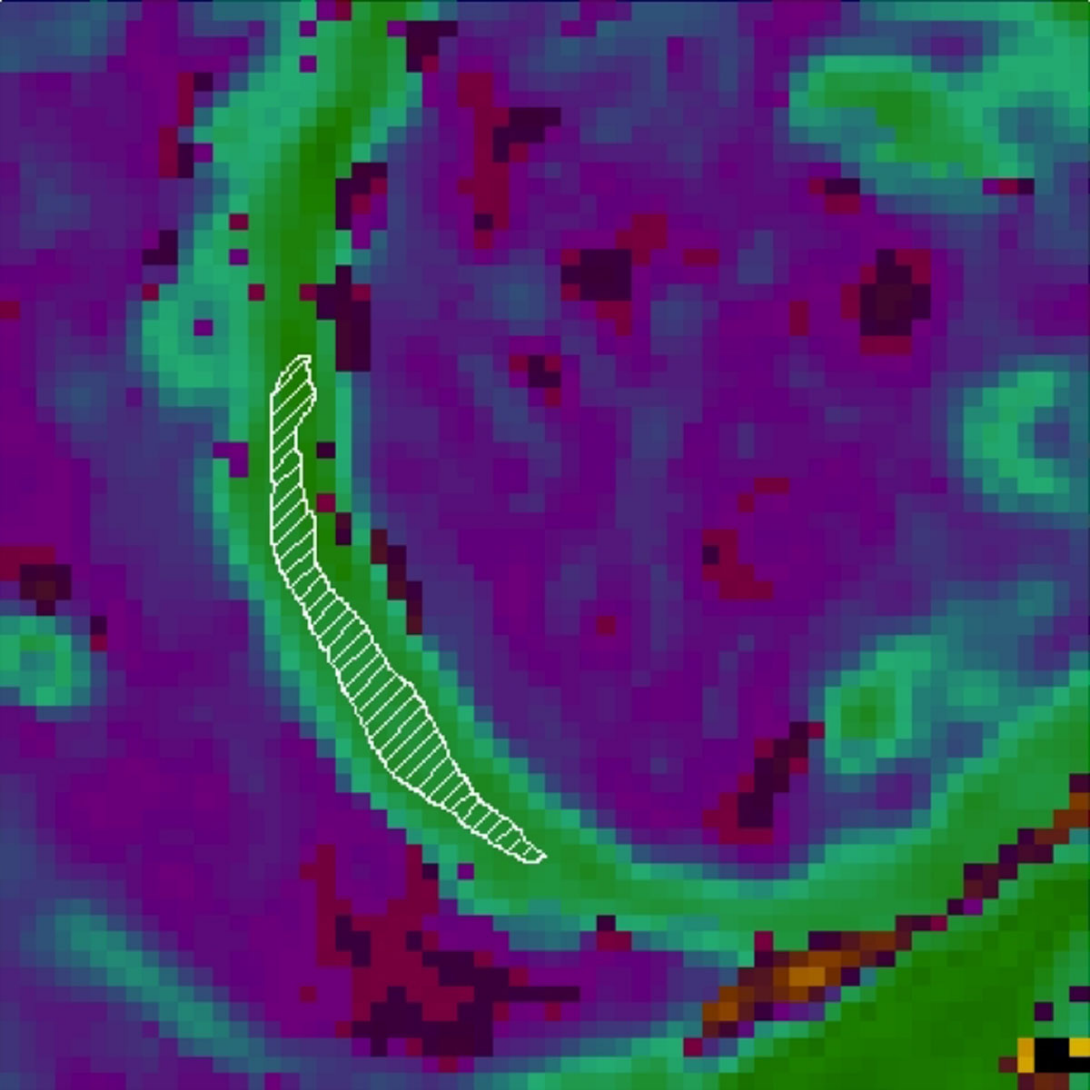
T1 map with outline of region of interest placed conservatively within the septal myocardium.

Fifty subjects (age 47 ± 7.4 years) underwent a routine CMR protocol on 3T clinical scanner with dual 32 channel coil and multi-transmit system. A single breath-hold hrMOLLI imaging was performed in an equatorial short axis slice prior and at selected intervals following administration of 0.2 mmol/kg gadolinium-DTPA contrast (10, 20, 40 minutes) (Figure1). Imaging parameters were FOV 320x320; TR/TE 3.2/1.57 ms, flip angle 50°, interpolated voxel size 0.9x0.9x8mm, phase encoding steps n=166, Trigger delay: 450 msec. T1 map values were obtained by 3 independent observers by placing the region of interest (ROI) within the septal myocardium (Figure 2). In patients with myocardial scar on late gadolinium enhancement, ROIs were derived from the remote areas only. A subset of healthy subjects (n=6) had a repeated native hrMOLLI imaging for interstudy reproducibility within 3 weeks of the original scan. Agreement between and within observers was examined by paired t-test and linear regressions.

## Results

T1 maps could be analysed in 94% of all images obtained. Intra-observer mean differences (MD) for T1 values were 1.3ms (95%CI:-12.4-15.4) for native scans and 0.7 ms (-7.9-12.3) for overall postcontrast scans, whereas interobserver MDs were 0.3 ms (-6.3 -5.3) and 0.1 (-3.4 to 4.2), respectively. We further demonstrate an excellent overall (pre- and postcontrast) intra-and interobserver agreement in T1 relaxation times (intraobserver: r= 0.94, p<0.0001; interobserver: R=0.91, p<0.0001). When assessed separately the postcontrast data showed better agreement within and between observers, respectively (r~0.94, p<0.0001 for both) than in the precontrast native scans (intraobserver r= 0.81, p <0.0001; interobserver r=0.79, p<0.001). We further revealed close agreements between repeated studies (r=0.91, p<0.001; MD: 2.4 msec (95%CI: -3.1-10.2).

## Conclusions

We demonstrate that imaging with hrMOLLI as a single breath-held scan pre and postcontrast administration provides a robust tool to reproducibly derive T1 relaxation times in clinical setting.

## Funding

NIHR British Research Centre.

